# Supplementary material for: Skeletonema marinoi Extracts and Associated Carotenoid Fucoxanthin Downregulate Pro-Angiogenic Mediators on Prostate Cancer and Endothelial Cells
Source: Cells. 2023 Mar 30;12(7):1053. doi: 10.3390/cells12071053 (PMC10093511; doi:10.3390/cells12071053)
Supplement: Supplementary file 1 [file cells-12-01053-s001.zip › cells-2062857-supplementary.pdf]

Table S1. Amount (in µg) of marine pigments in *Skeletonema marinoi* algae.

Skeletonema marinoi	Fucoxanthin (µg)	Diatoxanthin (µg)
Batch 1	0.286	0.066
Batch 2	0.320	0.020
Batch 3	0.239	0.045

Table S2. Sequence of primers used for qPCR studies.

Gene	Forward	Reverse
CXCR4	5'-CTCCTCTTTGTCATCAC-GCTTCC-3'	5'-GGATGAGGACACTGCTG-TAGAG-3'
CXCL8	5'-GAGAGTGATTGAGAGTGGAC-CAC-3'	5'-CACAACCCTCTGCACCCAG-TTT-3'
VEGF	5'-TTGCCTTGCTGCTCTACCTCCA-3'	5'-GATGGCAG-TAGCTGCGCTGATA-3'
TNFα	5′-CTCTTCTGCCTGCTGCACTTTG-3′	5′-ATGGGCTACAGGCTT-GTCACTC-3'
TGFβ1	5'-TACCTGAACCCGTGTTGCTCTC-3'	5'-GTTGCTGAGGTATCGCCAG-GAA-3'
TGFβ2	5'-AAGAAGCGTGCTTT-GGATGCGG-3'	5'-ATGCTCCAGCACAGAAGTT-GGC-3'
MMP2	5'-AGCGAGTG-GATGCCGCCTTTAA-3'	5'-CATTCCAGGCATCTGCGATGAG-3'
MMP9	5'- GCCACTACTGTGCCTTT-GAGTC-3'	5'-CCCTCAGAGAATCGCCAG-TACT-3'
TIMP1	5'-GGAGAGTGTCTGCGGA-TACTTC-3'	5'-CAGGTAGTGATGTGCAA-GAGTC-3'
TIMP2	5'-ACCCTCTGTGACTTCATCGTGC-3'	5'-GGAGATGTAGCACGG-GATCATG-3'
STAT3	5'- CTTTGAGACCGAGGTG-TATCACC -3'	5'- GGTCAGCATGTTGTAC-CACAGG -3'
IL-6	5'-AGACAGCCACTCAC-CTCTTCAG-3'	5'-TTCTGCCAGTGCCTCTTTGCTG-3'
VCAN	5'-TTGGACCTCAGGCGCTTTCTAC-3'	5'-GGATGACCAATTACAC-TCAAATCAC-3'
IL10	5'-TCTCCGAGATGCCTTCAG-CAGA-3'	5'-TCAGACAAGGCTTGG-CAACCCA-3'
IL1β	5'-CCACAGACCTTCCAGGA-GAATG-3'	5'-GTGCAGTTCAGTGATCG-TACAGG-3'
Angiogenin	5'-TGGCAACAAGCGCAG-CATCAAG-3'	5'-GCAAGTGGTGACCTGGAAA-GAAG-3'
CXCL5	5'-CAGACCACGCAAGGAG-TTCATC-3'	5'-TTCCTTCCCGTTCTTCAGGGAG-3'
PLGF	5'-GGCGATGAGAATCTGCAC-TGTG-3'	5'-ATTCGCAGCGAAC-GTGCTGAGA-3'
PDGF-BB	5'-GAGATGCTGAGTGACCAC-TCGA-3'	5'-GTCATGTTCAGGTCCAACTCGG-3'
ANGPT2	5'-ATTCAGCGACGTGAG-GATGGCA-3'	5'-GCACATAGCGTT-GCTGATTAGTC-3'
MMP1	5'-ATGAAGCAGCCCAGATGTG-GAG-3'	5'-TGGTCCACATCTGCTCTTGGCA-3'
GRO	5'-AGCTTGCCTCAATCCTGCATCC-3'	5'-TCCTTCAGGAACAGCCAC-CAGT-3'
Housekeeping		

β-actin	5'-CACCATTGG-CAATGAGCGGTTC-3'	5'-AGGTCTTTGCG-GATGTCCACGT-3'

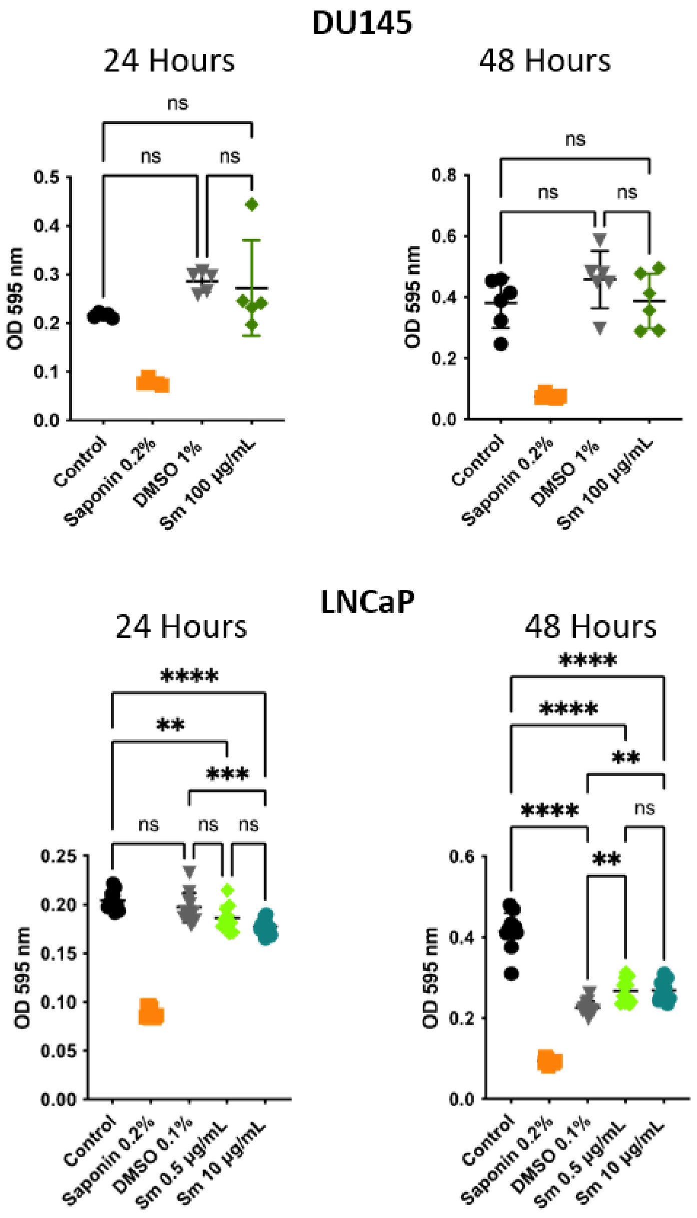

Figure S1. Effects of *S. marinoi* on PCa cell lines proliferation. The proliferation rate was measured by crystal violet assay (OD 595 nm). DU145 cells were treated with *Sm* at 100 µg/mL, 24 h and 48 h. LNCaP cells were treated with *Sm* at 0.5 and 10 µg/mL, 24 h and 48 h. DMSO was used as vehicle control. Results are shown as mean ± SEM, one-way ANOVA, ** $p < 0.01$, *** $p < 0.001$, **** $p < 0.0001$. (n = 5).

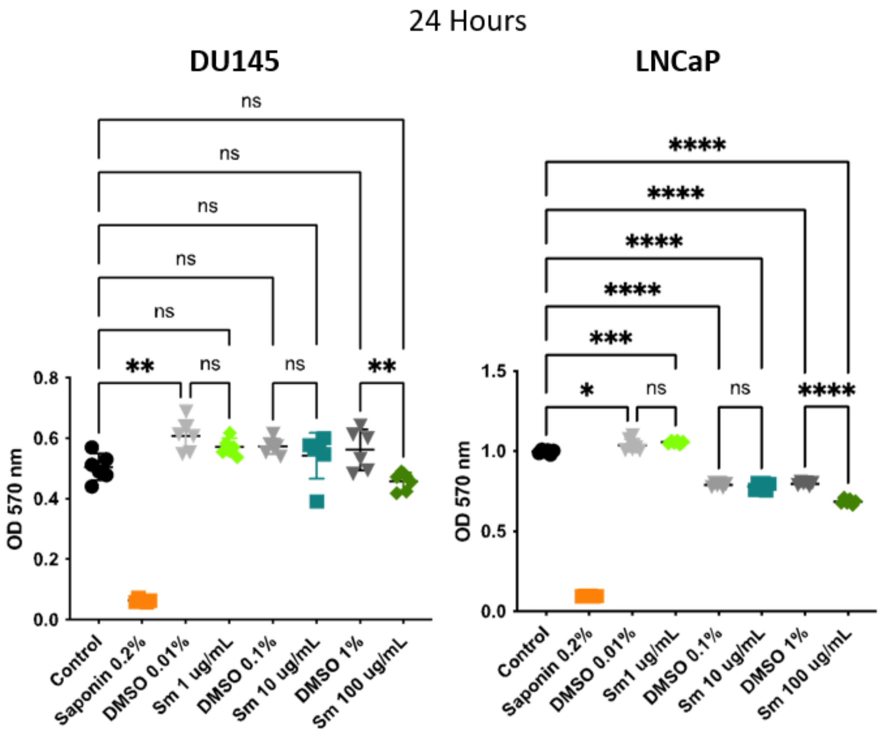

Figure S2. Effects of *S. marinoi* on PCa cell lines proliferation. The proliferation rate was measured by MTT (OD 570 nm). DU145 and LNCaP cells were treated with *Sm* at 1, 10 and 100 μg/mL, for 24 h. DMSO was used as vehicle control. Results are shown as mean ± SEM, one-way ANOVA, $^{*}p<0.05$, $^{**}p < 0.01$, $^{***}p < 0.001$, $^{****}p < 0.0001$. ($n = 5$).

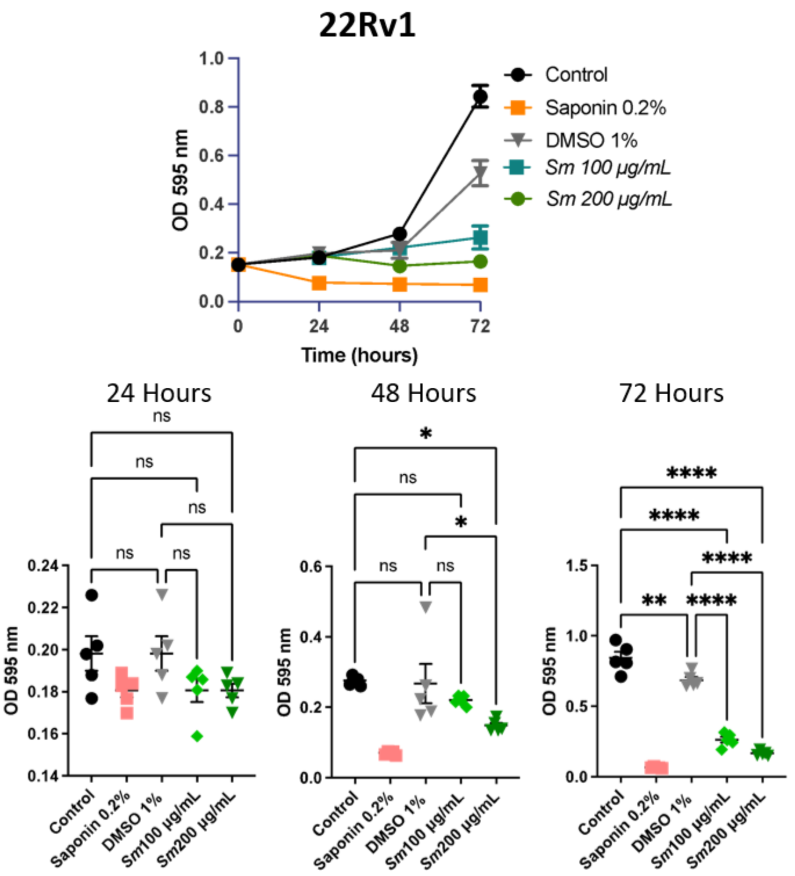

Figure S3. Effects of *S. marinoi* on 22Rv1 PCa cell lines proliferation. The proliferation rate was measured by crystal violet assay (OD 595 nm). 22Rv1 cells were treated with *Sm* at 100 µg/mL and 200 µg/mL, for 24 h, 48 h and 72 h. DMSO was used as vehicle control. Results are shown as mean ± SEM, one-way ANOVA, ns=not significant, * p < 0.05, ** p < 0.01, **** p < 0.0001 (*n* = 5).

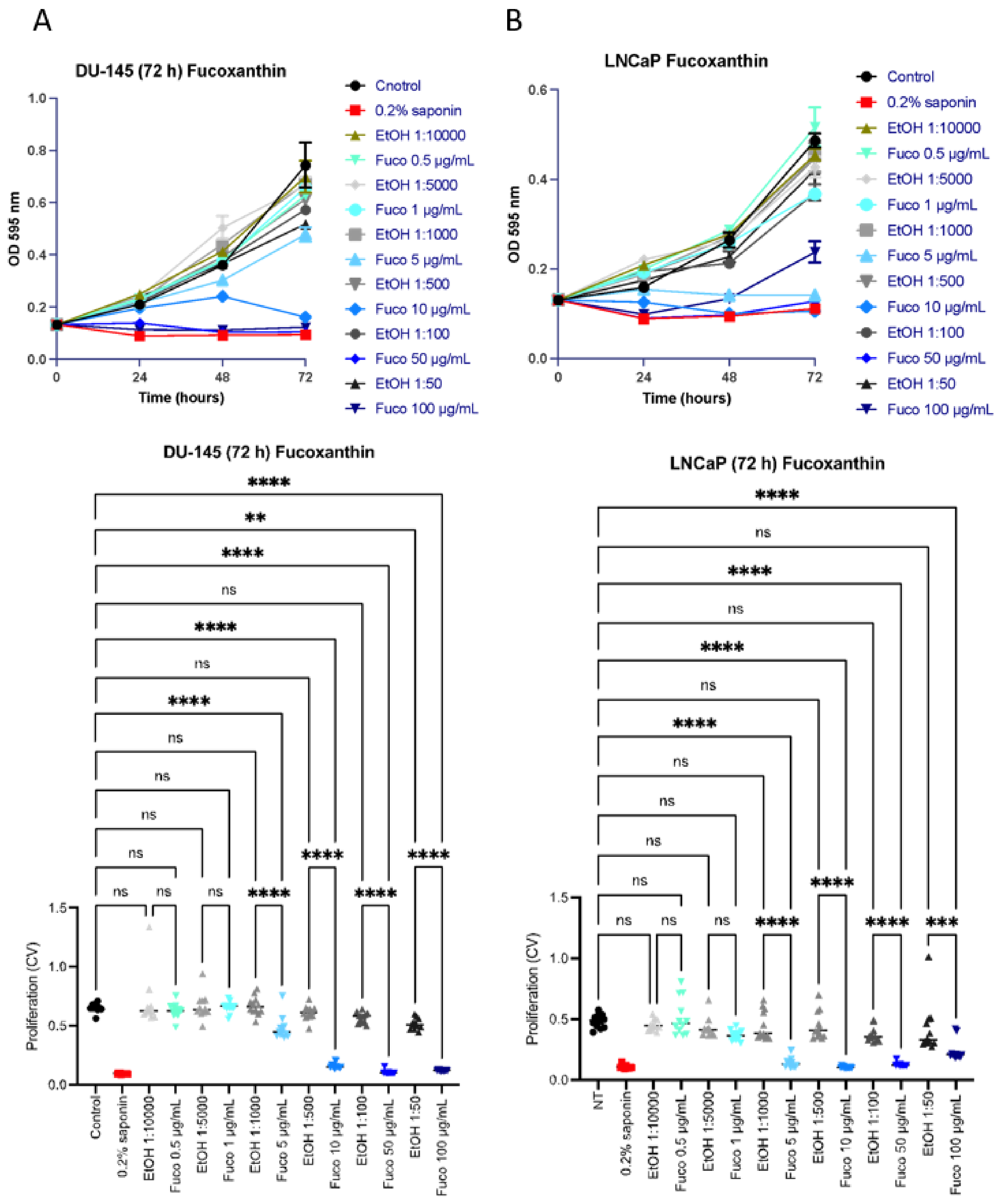

Figure S4. Effects of fucoxanthin on PCa cell lines proliferation. The proliferation rate was measured by crystal violet assay (OD 595 nm). DU-145 (**A**) and LNCaP (**B**) cells were treated with Fuco at 0.5, 1, 5, 10, 50, and 100 µg/mL for 24 h, 48 h and 72 h. DMSO was used as vehicle control. Results are shown as mean ± SEM, one-way ANOVA, ns=not significant, * p < 0.05, ** p < 0.01, **** p < 0.0001 (*n* = 5).

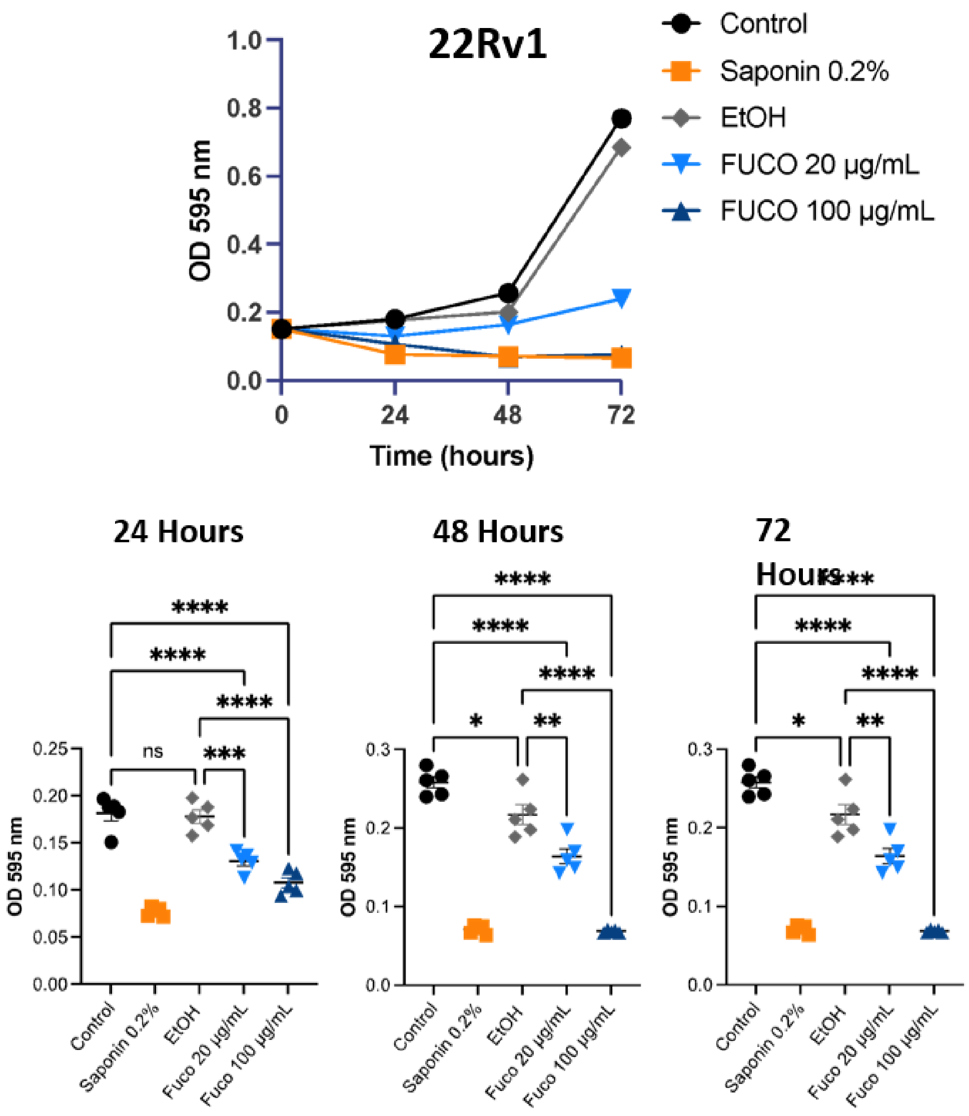

Figure S5. Effects of fucoxanthin on PCa cell lines proliferation. The proliferation rate was measured by crystal violet assay (OD 595 nm). 22Rv1 cells were treated with Fuco at 20 and 100 µg/mL. EtOH was used as vehicle control. Results are shown as mean ± SEM, one-way ANOVA, ns=not significant, ** $p < 0.01$, **** $p < 0.0001$ ($n = 5$).

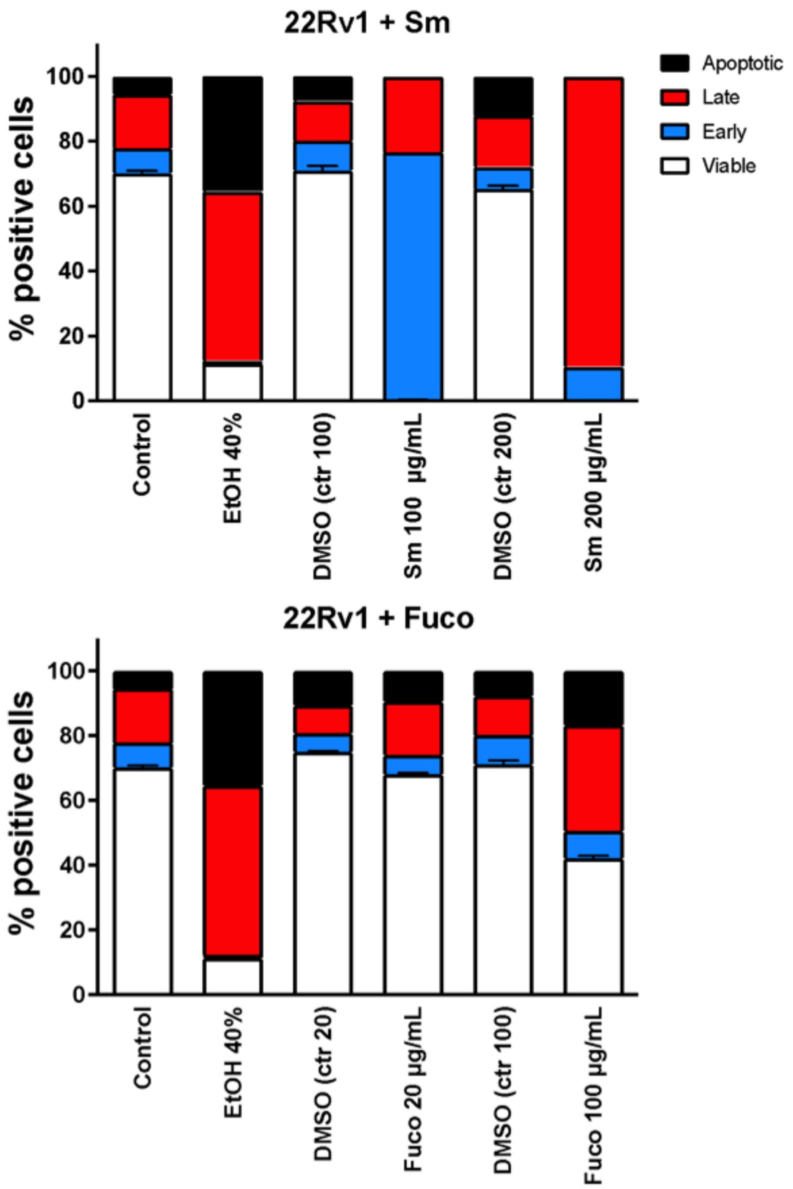

Figure S6. Effects of *Sm* extract and fucoxanthin in vitro on the induction of apoptosis in 22Rv1 PCa cell line. Cells were treated, for 24 h and 48 h, with *Sm* (100 and 200 µg/mL) and Fuco (20 and 100 µg/mL). The effects on induction of apoptosis were evaluated by flow cytometry (FACS analysis) following labeling with Annexin V/PI. Data were represented as bar graphs (n = 2). Control (not treated cells), EtOH (ethanol used at 40% as positive control), DMSO (vehicle control, used to dissolve *Sm* and Fuco).

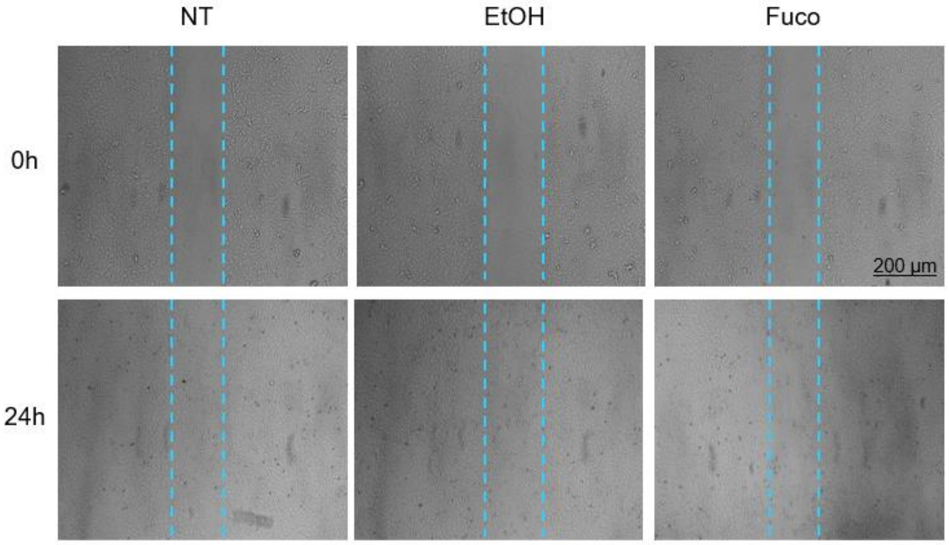

Figure S7. Effects of Fucoxanthin on HUVECs migration. Cells were treated with Fuco (20 µg/mL) or at corresponding concentration of EtOH (vehicle control). Cell migration was examined by wound healing assay at 0 h and 24 h. Fuco decreased migration of cells compared to control (NT, not treated). Images were taken at 4× magnification, representative images are shown. Scale bar: 200 µm (n = 6).

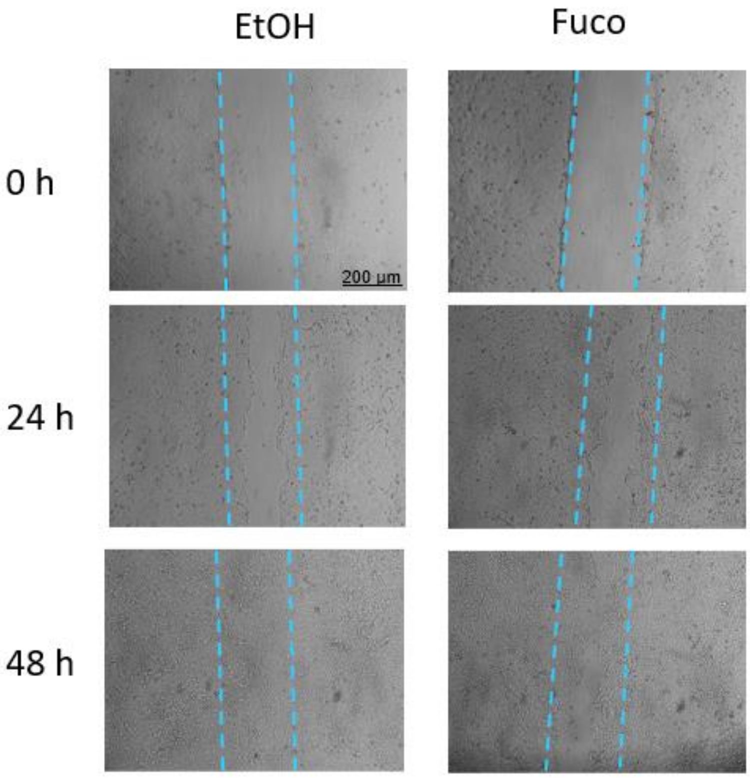

Figure S8. Effects of Fucoxanthin on DU145 cell line migration. Cells were treated with Fuco (20 µg/mL) or a corresponding concentration of EtOH (vehicle control). Cell migration was examined by wound healing assay at 0 h, 24 h and 48 h. Fuco decreased migration of cells compared to control (NT, not treated). Images were taken at 4x magnification, representative images are shown. Scale bar: 200 µm (n = 6).

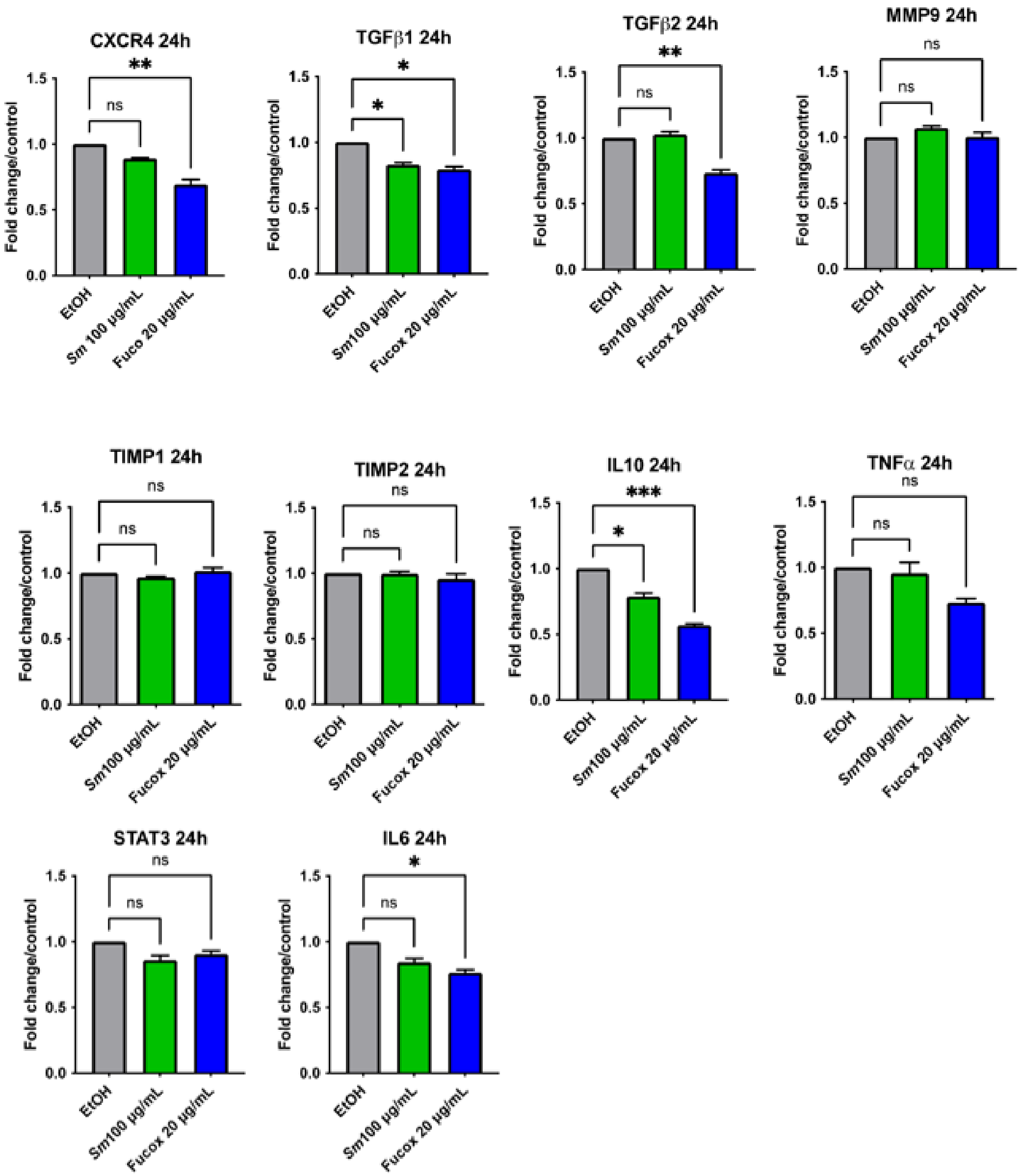

Figure S9. Gene expression profiling in PCa cells treated with *Sm* and marine pigment fucoxanthin. The ability of *Sm* (100 µg/mL) and Fuco (20 µg/mL) to inhibit expression in DU145 cells was determined, following 24 h of treatment, by qPCR. Data are shown as mRNA relative expression, normalized to β-actin, results are shown as mean ± SEM, one-way ANOVA, ns=not significant, * $p < 0.05$, ** $p < 0.01$, *** $p < 0.001$ ($n = 6$).

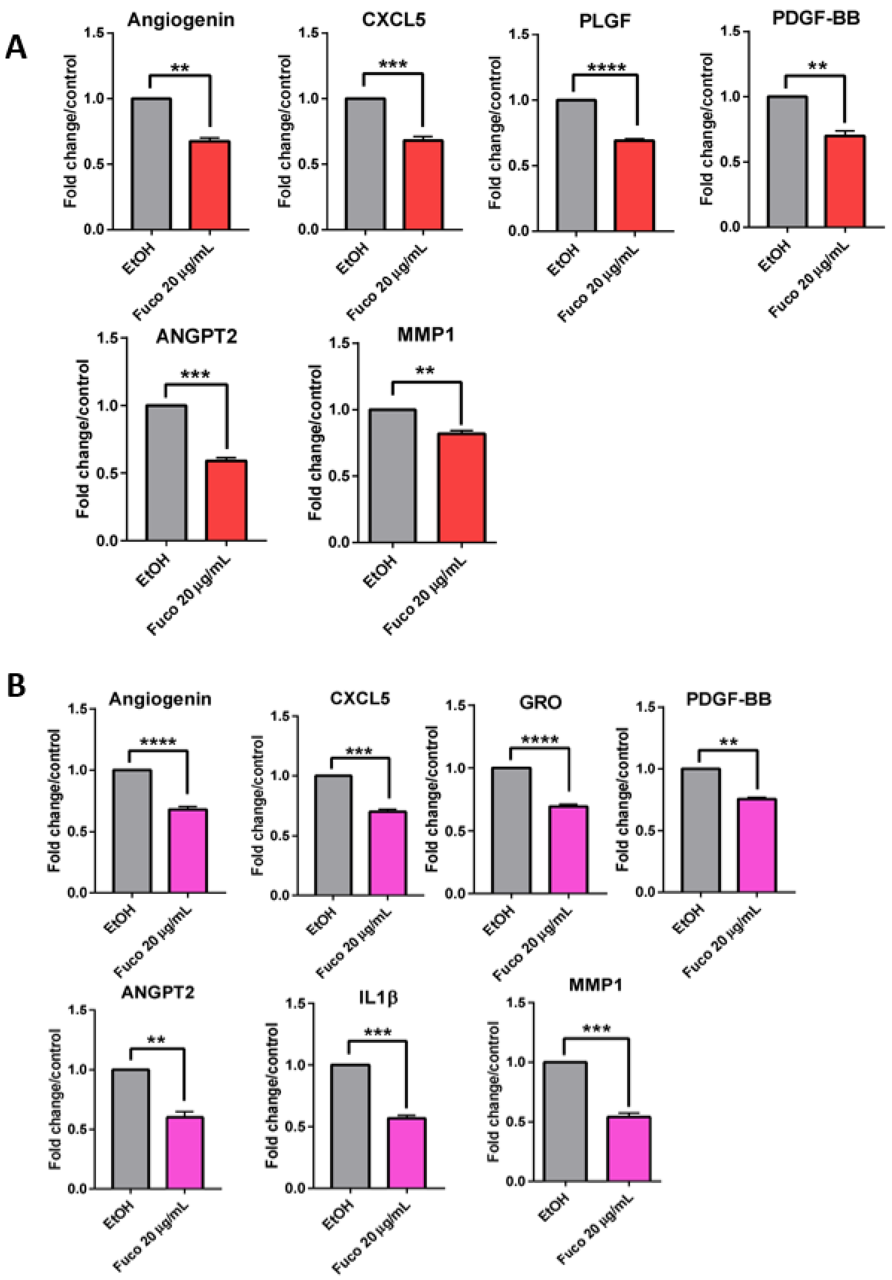

Figure S10. Angiogenesis gene expression profiling in HUVEC and DU145 cells treated with fucoxanthin. The ability of Fuco (20 µg/mL) to inhibit expression in HUVEC (A) and DU145 (B) cells was determined, following 6 h of treatment, by qPCR. Data are shown as mRNA relative expression, normalized to β-actin, results are shown as mean ± SEM, one-way ANOVA, ns=not significant, * $p < 0.05$, ** $p < 0.01$, *** $p < 0.001$ ($n = 6$).

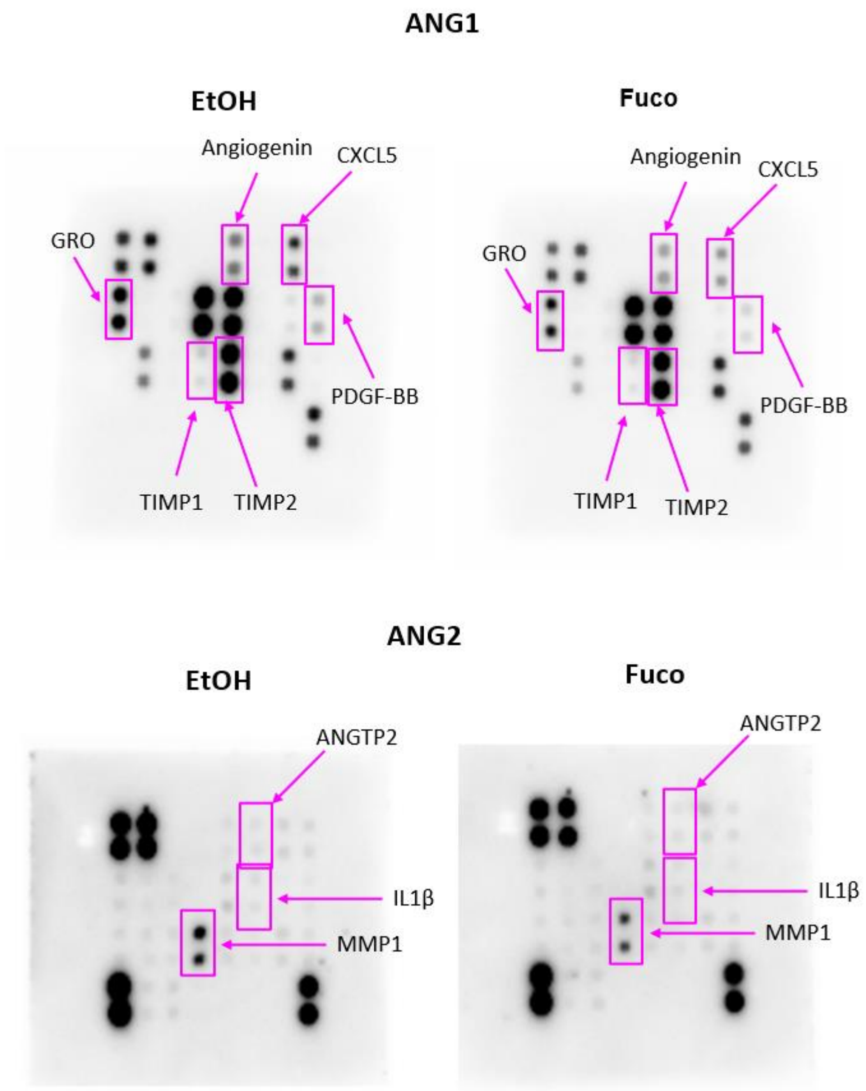

Figure S11. Protein expression profiling in PCa cells treated with fucoxanthin. Antibody arrays represent cytokines expression in DU145 cells after treatment with Fuco (20 μg/mL). EtOH was used as vehicle control. (*n* = 2). ANG1 and ANG2: Human Angiogenesis Array C1 and C2, respectively.
